# Phenylephrine Versus Noradrenaline for the Prevention and Treatment of Spinal-Induced Hypotension During Caesarean Section: A Literature Review

**DOI:** 10.7759/cureus.111632

**Published:** 2026-06-27

**Authors:** Manar A Alrashid, Jood H Hamdan, Soroush Ansari Lari

**Affiliations:** 1 Department of Management, University of Liverpool, Liverpool, GBR; 2 Department of Obstetrics and Gynaecology, Royal College of Surgeons in Ireland - Bahrain, Busaiteen, BHR; 3 Department of Medicine, Royal College of Surgeons in Ireland - Bahrain, Busaiteen, BHR

**Keywords:** caesarean section, hypotension, maternal haemodynamics, neonatal outcomes, noradrenaline, phenylephrine, spinal anaesthesia, vasopressors

## Abstract

Spinal anaesthesia is the most commonly used anaesthetic technique for caesarean section due to its rapid onset, reliability, and favourable safety profile. Despite these advantages, spinal-induced hypotension remains a common complication with potential adverse effects on maternal and fetal well-being, including nausea, vomiting, dizziness, reduced cardiac output, and impaired uteroplacental perfusion. Phenylephrine remains the guideline-supported first-line vasopressor for spinal-induced hypotension during caesarean delivery because of its effectiveness in maintaining maternal blood pressure and its favourable fetal acid-base profile, including a lower risk of fetal acidosis compared with ephedrine. However, it may cause reflex bradycardia and reduced maternal cardiac output. Norepinephrine (noradrenaline) has emerged as a promising alternative because its α-adrenergic effects, combined with modest β1 activity, may better preserve maternal heart rate and cardiac output. A narrative literature review was conducted using PubMed/MEDLINE, Scopus, and Google Scholar. Studies were included if they evaluated maternal haemodynamic outcomes, adverse maternal effects, neonatal outcomes, or vasopressor efficacy during caesarean delivery under spinal anaesthesia. Recent trials and meta-analyses suggest that noradrenaline provides comparable efficacy to phenylephrine, with similar neonatal outcomes and potential maternal haemodynamic advantages. However, phenylephrine remains the current guideline-supported first-line agent, while noradrenaline requires further clarification regarding optimal dosing, safety, and routine first-line use. This review evaluates phenylephrine and noradrenaline for both prophylaxis and rescue treatment of spinal-induced hypotension during caesarean section.

## Introduction and background

Caesarean section is one of the most frequently performed surgeries in obstetric practice, and spinal anaesthesia is the preferred technique for elective caesarean delivery because it provides rapid, reliable anaesthesia while avoiding many of the risks associated with general anaesthesia [1,2]. Despite these advantages, spinal-induced hypotension remains a prevalent challenge, affecting the majority of untreated patients due to acute sympathetic blockade [3,4].

Spinal-induced hypotension primarily occurs from sympathetic blockade leading to arterial and venous vasodilation, reduced systemic vascular resistance, venous pooling, and decreased venous return [1]. The reported incidence of hypotension in women under spinal anaesthesia varies depending on the definition used, and without prophylactic measures, it can affect the majority of women undergoing a caesarean delivery [3]. Maternal hypotension is associated with nausea, vomiting, dizziness, and discomfort, while severe or prolonged hypotension may reduce uteroplacental perfusion and contribute to adverse neonatal acid-base changes [1,4]. Therefore, maintaining maternal blood pressure is a key objective of obstetric anaesthetic management.

Vasopressors are key to both prevention and treatment of spinal-induced hypotension. Previously, ephedrine was favoured because of concerns that pure alpha-adrenergic agonists could reduce uteroplacental blood flow [5]. However, subsequent evidence showed that phenylephrine effectively restores maternal blood pressure and improves fetal acid-base status compared to ephedrine [6,7]. As a result, phenylephrine has become the preferred vasopressor in many obstetric anaesthetic guidelines and is widely used in current practice [2,8].

While phenylephrine is highly effective at counteracting this via selective α1-adrenergic vasoconstriction, its tendency to induce reflex bradycardia can significantly drop maternal cardiac output [9,10]. Preserving maternal cardiac output, rather than merely chasing a macrovascular blood pressure target, is clinically vital; it maintains systemic tissue perfusion and ensures stable, uncompromised uteroplacental blood flow, minimizing the risk of hidden fetal hypoperfusion.

Noradrenaline is emerging as a promising alternative vasopressor in obstetric anaesthesia. Unlike phenylephrine, noradrenaline has strong α-adrenergic activity with modest β1-adrenergic effects, meaning it can maintain blood pressure while better preserving heart rate and cardiac output [10,11]. Several randomised controlled trials have reported comparable efficacy between noradrenaline and phenylephrine, with less bradycardia and more favourable maternal haemodynamic effects [12-14]. Recent systematic reviews and meta-analyses have also reported broadly similar maternal and neonatal outcomes between the two agents. However, there is a clear knowledge gap in current literature: while the immediate physiological benefits of noradrenaline are well-documented, we lack large-scale trials in high-risk cohorts (such as pre-eclamptic or growth-restricted pregnancies) and have no data on long-term neonatal neurodevelopmental outcomes [15-17]. Consequently, it is unclear whether noradrenaline represents a true clinical advancement or simply an alternative hemodynamic pathway.

This literature review critically examines the current evidence comparing phenylephrine and noradrenaline for the prevention and treatment of spinal-induced hypotension during caesarean section. It particularly focuses on maternal haemodynamic outcomes, adverse effects, neonatal well-being, limitations of the current evidence, and implications of current findings for future obstetric anaesthetic practice.

## Review

Methods

This literature review was done by identifying studies through searches of PubMed/MEDLINE, Scopus, and Google Scholar using combinations of the terms "caesarean section", "spinal anaesthesia", "maternal hypotension", "phenylephrine", "norepinephrine", "noradrenaline", and "vasopressors".

Searches were limited to English language studies involving human participants and focused on obstetric populations undergoing caesarean delivery under spinal anaesthesia. Randomised controlled trials, systematic reviews, meta-analysis and relevant clinical guidelines were eligible for inclusion. Studies were excluded if they involved non-obstetric populations, alternative anaesthetic techniques, animal models, or did not report maternal or neonatal outcomes relevant to vasopressor use.

Titles and abstracts were screened for relevance, and full-text articles were reviewed where appropriate. Studies were included if they evaluated vasopressor efficacy, maternal haemodynamic outcomes, maternal adverse effects, neonatal outcomes, or clinical guideline recommendations relating to the use of phenylephrine or noradrenaline during caesarean delivery under spinal anaesthesia. Evidence was synthesised narratively to compare the efficacy and safety profiles of the two vasopressors, identify strengths and limitations within the current evidence base, and explore implications for clinical practice and future research.

No quantitative synthesis or meta-analysis was undertaken. Evidence was summarised narratively by outcome domain, and heterogeneity in dosing, administration method, hypotension definitions, outcome measures, and study populations was considered when interpreting the findings.

Evolution of vasopressors

The transition from ephedrine to phenylephrine as the preferred vasopressor in obstetric anaesthesia was largely driven by accumulating evidence demonstrating improved neonatal outcomes. A systematic review conducted by Lee et al. compared ephedrine and phenylephrine for the management of spinal anaesthesia-induced hypotension during caesarean delivery [5]. It was concluded that phenylephrine was as effective as ephedrine in maintaining maternal blood pressure and was not associated with adverse neonatal outcomes. These findings challenged the long-held belief that ephedrine was inherently safer for the fetus and encouraged clinicians to reconsider established practice. However, the review was limited by the quality of the available evidence. Many of the included studies had small sample sizes, used different dosing regimens, and employed varying definitions of hypotension. Such heterogeneity limits the ability to perform direct comparisons and reduces the overall strength of the conclusions [5]. Furthermore, neonatal outcomes were often assessed using Apgar scores, which may lack sensitivity to detect subtle differences in fetal well-being [5]. Despite these limitations, the review represented an important turning point in obstetric vasopressor research by highlighting the lack of evidence supporting the superiority of ephedrine [5].

Further evidence supporting phenylephrine emerged from a randomised controlled trial by Ngan Kee et al. [6], which compared combinations of phenylephrine and ephedrine infusions during caesarean delivery. The study demonstrated that phenylephrine-based regimens were associated with higher umbilical arterial pH values and less fetal acidosis than ephedrine-based regimens while maintaining effective maternal blood pressure control. The use of umbilical cord blood gas analysis strengthened the study as it provided a more objective measure of neonatal well-being than Apgar scores alone [6]. However, the study included only healthy women undergoing elective caesarean sections, limiting the generalisability of the findings to emergency procedures and high-risk pregnancies [6]. In addition, the relatively modest sample size reduced the statistical power to detect uncommon maternal or neonatal adverse events [6]. Despite these limitations, the findings provided some of the most compelling evidence supporting phenylephrine over ephedrine with respect to fetal acid-base status.

Similar findings were reported in a subsequent randomised controlled trial by Ngan Kee et al. [7], which evaluated vasopressor use during non-elective caesarean section. The study demonstrated that phenylephrine effectively treated maternal hypotension without adversely affecting neonatal outcomes, thereby extending previous evidence beyond the elective setting and supporting its use in more urgent scenarios. However, the study still excluded many high-risk obstetric patients, including those with severe maternal comorbidities or significant fetal compromise. As a result, caution should be exercised when extrapolating the findings to all obstetric populations [7].

Although phenylephrine demonstrated clear advantages over ephedrine with respect to fetal acid-base status, concerns regarding its maternal haemodynamic effects began to emerge. Dyer et al. [9] investigated the haemodynamic effects of commonly used vasopressors during caesarean delivery and found that phenylephrine was associated with reductions in maternal heart rate and cardiac output secondary to reflex bradycardia. This finding was clinically significant as maintenance of blood pressure does not necessarily equate to optimal cardiovascular performance or tissue perfusion. However, the study was conducted in a controlled elective setting and relied on haemodynamic monitoring techniques that may not fully reflect real-world obstetric practice. Furthermore, the clinical significance of these transient reductions in cardiac output remains uncertain, particularly in healthy parturients, which limits the applicability of the findings to the broader population.

Overall, the available evidence suggests that phenylephrine replaced ephedrine primarily because of its association with improved fetal acid-base outcomes and efficacy in maintaining maternal blood pressure. However, many of the studies underpinning this shift were conducted in relatively low-risk populations and were not powered to assess uncommon adverse outcomes [6,7]. Additionally, the increasing recognition that phenylephrine may reduce maternal heart rate and cardiac output prompted questions regarding whether a more favourable vasopressor could better preserve maternal haemodynamics [9,10]. These concerns ultimately stimulated interest in noradrenaline as a potential alternative, paving the next stage in the evolution of vasopressor research within obstetric anaesthesia.

Pharmacology of phenylephrine and noradrenaline

The growing interest in noradrenaline as an alternative vasopressor for spinal-induced hypotension is largely driven by its distinction in adrenergic receptor activity compared to phenylephrine. Phenylephrine acts predominantly as an α1-adrenergic agonist, increasing systemic vascular resistance through peripheral vasoconstriction [9,10]. In contrast, noradrenaline possesses both potent α1-adrenergic activity and modest β1-adrenergic effects, which can contribute to greater heart rate and cardiac output preservation [10,11]. These pharmacological differences have important implications for maternal haemodynamic responses during caesarean delivery under spinal anaesthesia [9,10].

The limitations of phenylephrine became increasingly apparent as studies began examining cardiovascular parameters beyond blood pressure alone. Dyer et al. demonstrated that, although phenylephrine effectively restored arterial pressure following spinal anaesthesia, it was associated with reductions in maternal heart rate and cardiac output [9]. The authors suggested that reflex bradycardia resulting from increased vascular resistance may partially explain these findings. Although the study highlighted an important physiological limitation of phenylephrine, its findings should be interpreted cautiously. The study population consisted primarily of healthy women undergoing elective caesarean section, limiting applicability to higher-risk obstetric populations. Furthermore, despite reductions in cardiac output, both maternal and neonatal outcomes remained favourable, resulting in uncertainty regarding the clinical significance of these haemodynamic changes.

Evidence supporting the potential advantages of noradrenaline emerged from the randomised controlled trial conducted by Ngan Kee et al. [10]. In this study, women receiving noradrenaline experienced significantly fewer episodes of bradycardia than those receiving phenylephrine while maintaining comparable systolic blood pressure control. Importantly, cardiac output was better preserved in the noradrenaline group, supporting the hypothesis that β1-adrenergic activity may mitigate the reduction in heart rate observed with phenylephrine. As one of the first studies directly comparing the two vasopressors in obstetric anaesthesia, this study provided an important physiological basis for further evaluation of noradrenaline. However, the study was conducted at a single centre and involved only 104 women, limiting its power to detect differences in neonatal outcomes or rare adverse events. Although the results were statistically significant, their external validity remains limited.

Further evidence was provided by Ngan Kee, who conducted a dose-response study to determine equipotent doses of phenylephrine and noradrenaline [11]. The study estimated a potency ratio of approximately 13:1, indicating that noradrenaline achieves comparable vasopressor effects at substantially lower doses. This represented an important methodological advancement as earlier studies often used empirically selected dosing regimens, limiting direct comparisons. Nevertheless, dose-response studies primarily evaluate pharmacological potency rather than clinical effectiveness. Though the study improved understanding of vasopressor dosing, it did not establish whether one agent resulted in superior maternal or neonatal outcomes.

Similar findings were reported by Cho et al., who compared intermittent boluses of noradrenaline and phenylephrine during elective caesarean delivery [14]. The authors reported significantly higher heart rates and cardiac output measurements in women receiving noradrenaline, while blood pressure control remained comparable between groups. These findings support the theoretical advantage conferred by noradrenaline's β1-adrenergic activity. However, the study included a relatively small sample and utilised non-invasive cardiac output monitoring, which is less accurate than invasive haemodynamic measurements; thus, the magnitude of the observed differences should be interpreted cautiously.

Although these studies suggest physiological advantages associated with noradrenaline, the evidence remains largely focused on surrogate haemodynamic endpoints. Most investigations have evaluated blood pressure, heart rate, cardiac output, and incidence of bradycardia rather than clinically meaningful outcomes, such as severe maternal morbidity, neonatal complications, intensive care admission, or long-term neonatal well-being [10,12-14]. This limitation is particularly important because improvements in physiological parameters do not necessarily translate into improved clinical outcomes [15,16].

The limitations of the current evidence are reflected in recent systematic reviews. Liu et al. concluded that noradrenaline was associated with a lower incidence of maternal bradycardia compared with phenylephrine while maintaining similar efficacy in preventing hypotension [15]. However, the authors noted substantial heterogeneity between studies, particularly regarding vasopressor dosing, administration methods, and definitions of hypotension. Similarly, Ahmed et al. reported comparable maternal and neonatal outcomes between the two vasopressors despite modest haemodynamic advantages favouring noradrenaline [16]. These findings suggest that, while the pharmacological rationale for noradrenaline is compelling, evidence supporting clinically significant superiority remains limited.

Overall, current evidence demonstrates that the principal pharmacological advantage of noradrenaline lies in its ability to maintain blood pressure while better preserving maternal heart rate and cardiac output. However, much of this evidence is derived from relatively small studies conducted in healthy women undergoing elective caesarean section and focuses primarily on surrogate haemodynamic outcomes. Therefore, larger studies assessing clinically relevant maternal and neonatal outcomes are needed before definitive conclusions can be made regarding the superiority of noradrenaline over phenylephrine, despite supportive pharmacological data [15-17].

Maternal haemodynamic outcomes

Maternal haemodynamic stability is a key objective of the management of spinal-induced hypotension during caesarean section. Commonly reported outcomes include blood pressure control, heart rate, bradycardia, cardiac output, and the need for rescue vasopressors. Although noradrenaline has gained interest because of its ability to preserve heart rate and cardiac output, the evidence is not uniformly supportive of replacing phenylephrine. Much of the literature suggests that the two agents provide similar blood pressure control [10,12,13,15,16], while the main advantage of noradrenaline lies in surrogate haemodynamic outcomes rather than clearly demonstrated improvements in maternal or neonatal clinical outcomes [15-17].

Blood Pressure 

The primary purpose of vasopressor administration during caesarean section under spinal anaesthesia is the prevention and treatment of maternal hypotension. Evidence consistently demonstrates that both phenylephrine and noradrenaline are effective in maintaining blood pressure [10,12,13,15,16]. Ngan Kee et al. found that noradrenaline maintained systolic blood pressure as effectively as phenylephrine during elective caesarean delivery [10]. Similarly, Vallejo et al. reported no significant difference in hypotension prevention between continuous infusions of phenylephrine and noradrenaline [12]. This suggests that noradrenaline is not inferior to phenylephrine in maintaining arterial pressure [10,12].

However, the evidence does not clearly show that noradrenaline is superior for blood pressure control. Sharkey et al., using intermittent bolus doses, found comparable effectiveness between the two agents in preventing and treating hypotension [13]. This is important because it challenges the assumption that noradrenaline should replace phenylephrine simply because it has a more favourable receptor profile. Meta-analytic evidence supports this cautious interpretation. Liu et al. found that noradrenaline and phenylephrine had similar efficacy in preventing maternal hypotension [15], while Ahmed et al. also reported no significant difference in maternal hypotension across treatment groups [16].

The main limitation of these findings is the heterogeneity within the literature. Studies vary in respect to vasopressor dosing, route of administration, use of prophylactic versus rescue treatments, and definition of hypotension. For example, some trials define hypotension as a systolic blood pressure below 80% of baseline, while others use fixed systolic blood pressure thresholds. Such variation makes it difficult to determine whether observed differences between studies are due to the drug itself or to differences in trial design [15,16]. Therefore, the strongest conclusion is that noradrenaline appears equivalent, rather than superior, to phenylephrine for blood pressure control [15,16].

Heart Rate and Bradycardia

Preservation of maternal heart rate represents the most consistently reported hemodynamic advantage of noradrenaline over phenylephrine. Phenylephrine acts mainly as an α1-adrenergic agonist and may cause reflex bradycardia following peripheral vasoconstriction [9,10]. In contrast, noradrenaline has α-adrenergic activity with weak β1-adrenergic effects, which may contribute to better preservation of heart rate [10,11].

The physiological advantage has been demonstrated across several clinical studies. Ngan Kee et al. reported significantly lower incidence of bradycardia in women receiving noradrenaline compared with phenylephrine [10]. Similar findings were reported by Vallejo et al., who observed improved heart rate preservation with noradrenaline infusion [12], while Sharkey et al. reported fewer bradycardic episodes with noradrenaline boluses [13]. These findings have been reinforced by systematic reviews. Liu et al. concluded that noradrenaline reduced the incidence of maternal bradycardia compared with phenylephrine [15], a finding Ahmed et al. subsequently supported [16]. However, this advantage should be interpreted critically. In many studies, bradycardia was a physiological endpoint rather than a clinically harmful event. Episodes were typically transient and treatable and rarely associated with adverse maternal or neonatal outcomes [10,12,13]. Moreover, in healthy women undergoing elective caesarean section, phenylephrine-induced bradycardia may be of less clinical relevance because cardiovascular reserve is generally preserved. Therefore, while noradrenaline is clearly associated with less bradycardia, there is insufficient evidence to conclude that this translates into improvements in clinical outcomes [15,16].

Cardiac Output

Cardiac output is an important parameter, as blood pressure alone may not accurately reflect maternal cardiovascular function [9,10]. While phenylephrine effectively restores blood pressure by increasing systemic vascular resistance, this may be accompanied by a reduction in heart rate and cardiac output. Dyer et al. demonstrated that phenylephrine was associated with reductions in maternal heart rate and cardiac output, supporting concerns that phenylephrine may produce a less favourable haemodynamic profile despite effective blood pressure control [9].

Ngan Kee et al. provided further evidence that noradrenaline may better preserve cardiac output while maintaining blood pressure control than phenylephrine [10], and similar findings were observed by Cho et al. [14]. These findings are pharmacologically plausible because noradrenaline has weak β1-adrenergic activity, which may support myocardial contractility and heart rate [10,11].

However, evidence supporting superior cardiac output preservation is limited by several factors. Firstly, many studies used non-invasive cardiac output monitoring, which may be less accurate than invasive techniques. Second, most trials were relatively small and conducted in low-risk elective caesarean populations. Third, although cardiac output was better preserved with noradrenaline, studies have not consistently shown that this translates into better maternal or neonatal clinical outcomes. For this reason, improved cardiac output should be viewed as a potentially important physiological advantage rather than definitive evidence of clinical superiority [15,16].

Requirement for Rescue Vasopressors

The requirement for rescue vasopressors is a useful practical measure because it reflects how well a prophylactic regimen prevents breakthrough hypotension. Available studies generally show similar rescue vasopressor requirements between phenylephrine and noradrenaline. Vallejo et al. found no significant difference in the need for rescue intervention between the two groups [12]. Sharkey et al. also reported comparable effectiveness when both agents were used as intermittent boluses [13].

This equivalence is important because it supports continued use of phenylephrine in routine practice [12,13]. If noradrenaline does not consistently reduce the need for rescue vasopressors, then its main advantage remains physiological rather than practical. However, interpretation is limited by differences in study protocols. Some trials used continuous prophylactic infusions, others used intermittent boluses, and some allowed rescue medication according to different thresholds. As a result, it is difficult to draw a firm conclusion about whether one agent offers superior real-world reliability.

Reactive Hypertension and Haemodynamic Overshoot

Reactive hypertension is another important haemodynamic outcome because excessive vasoconstriction may cause maternal discomfort and potentially reduce uteroplacental perfusion. Some meta-analytic evidence suggests that noradrenaline may be associated with less reactive hypertension than phenylephrine [16]. This may reflect the lower doses used or the more balanced haemodynamic profile of noradrenaline.

However, reactive hypertension is inconsistently defined across studies and is not always reported as a primary outcome. Therefore, while this finding may favour noradrenaline, the strength of evidence is weaker than for bradycardia. In addition, phenylephrine remains highly titratable and familiar to anaesthetists, which may reduce the risk of clinically significant hypertension when used appropriately.

Overall Comparison of Maternal Haemodynamic Outcomes 

Overall, the available evidence suggests that noradrenaline and phenylephrine are broadly equivalent in maintaining maternal blood pressure during caesarean section under spinal anaesthesia. The principal advantages of noradrenaline are improved preservation of heart rate and cardiac output, which results in lower rates of maternal bradycardia. However, these benefits are largely confined to surrogate hemodynamic outcomes, and current evidence has not demonstrated clear superiority with respect to clinically important maternal or neonatal endpoints. Moreover, noradrenaline should be considered as an alternative rather than a replacement, as supported by extensive clinical studies.

Maternal side effects and patient comfort

While haemodynamic stability remains the primary objective of vasopressor therapy during caesarean section, patient-centred outcomes are also important when evaluating vasopressor effectiveness. Maternal symptoms, such as nausea, vomiting, shivering, and intraoperative discomfort, can significantly affect the childbirth experience and are commonly used as secondary outcomes in studies comparing phenylephrine and noradrenaline. Although noradrenaline has demonstrated favourable haemodynamic characteristics, evidence regarding symptomatic maternal outcomes is limited. 

Nausea

Nausea is among the most frequently reported complications during caesarean delivery under spinal anaesthesia and is strongly associated with maternal hypotension [1,4]. Reduced cerebral and gastrointestinal perfusion secondary to inadequate blood pressure control is considered to be a major contributing factor [1,4]. Consequently, effective prevention of hypotension should theoretically reduce the incidence of nausea regardless of the vasopressor used.

Several randomised controlled trials have compared rates of maternal nausea between phenylephrine and noradrenaline. Ngan Kee et al. found no significant difference in nausea incidence despite improved haemodynamic parameters in the noradrenaline group [10]. Similar findings were reported by Vallejo et al. and Sharkey et al., both of whom observed comparable rates of nausea between treatment groups [12,13]. These findings suggest that, once adequate blood pressure control is achieved, the choice of vasopressor has little influence on the occurrence of nausea [10,12,13,15,16].

This conclusion is supported by meta-analytic evidence. Liu et al. found no significant difference in maternal nausea between noradrenaline and phenylephrine [15], while Ahmed et al. similarly reported equivalent rates across pooled analyses [16]. These findings indicate that the haemodynamic advantages associated with noradrenaline do not appear to cause a measurable reduction in maternal nausea.

However, several limitations should be considered when interpreting the findings due to the subjective nature of nausea. It is influenced by multiple factors, including anxiety, surgical manipulation, opioid administration, and individual susceptibility. In addition, nausea is often reported as a secondary outcome, meaning many studies may be underpowered to detect small but clinically relevant differences. Therefore, although current evidence suggests equivalence, subtle advantages of one vasopressor cannot be completely excluded [15,16].

Vomiting

Vomiting is an important cause of maternal discomfort during caesarean delivery and is commonly assessed alongside studies of vasopressor therapy. Current evidence from both randomised controlled trials and pooled analyses suggests no meaningful difference in vomiting incidence between phenylephrine and noradrenaline. Similar rates have been reported by Ngan Kee et al. [10], Vallejo et al. [12], and Sharkey et al. [13], with these findings subsequently validating recent systematic reviews [15,16].

Nevertheless, interpretation of these findings is limited by inconsistencies in outcome reporting, as some studies combine nausea and vomiting into a composite outcome. Furthermore, vomiting occurs relatively infrequently when effective prophylactic vasopressors are used, reducing the ability of the individual studies to detect small differences between treatments. Therefore, the current evidence suggests that phenylephrine and noradrenaline are broadly equivalent with respect to vomiting prevention [15,16].

Shivering

Shivering is another common maternal symptom during caesarean section under spinal anaesthesia and may contribute to discomfort, increased oxygen consumption, and difficulty with physiological monitoring [18]. The relationship between vasopressor choice and shivering remains less well established than for nausea and vomiting.

Most early trials comparing phenylephrine and noradrenaline did not evaluate shivering as a primary outcome. Consequently, evidence remains limited. However, some recent studies have suggested that noradrenaline may be associated with lower rates of shivering [18], potentially due to improved maintenance of peripheral vascular tone and thermoregulation. Despite this, findings remain inconsistent and have not been uniformly replicated across studies.

A major limitation of the evidence is that shivering is often reported incidentally rather than being actively assessed using validated scoring systems. In addition, factors such as operating theatre temperature, intravenous fluid warming, and patient characteristics may influence shivering independently of vasopressor choice. Therefore, current evidence is insufficient to conclude that either vasopressor provides a meaningful advantage regarding shivering prevention.

Maternal Comfort

Beyond individual symptoms, a small number of studies have evaluated broader measures of maternal comfort and satisfaction. However, the outcomes are infrequently reported and are often assessed using non-standardised tools to measure, which limits comparison with other studies. The available evidence suggests similar levels of patient satisfaction among women receiving phenylephrine and noradrenaline [12,13].

This finding highlights the patient-reported outcomes contrast with the hemodynamic advantages frequently reported for noradrenaline. This suggests that modest improvements in physiological parameters may not necessarily influence the overall maternal experience of caesarean delivery. Thus, although noradrenaline may offer certain haemodynamic benefits, current evidence does not demonstrate a clear advantage in terms of maternal comfort or satisfaction.

Overall Comparison of Maternal Side Effects and Patient Comfort

Overall, the available evidence suggests that phenylephrine and noradrenaline are associated with similar maternal side effect profiles during caesarean delivery under spinal anaesthesia. Rates of nausea and vomiting appear comparable between agents, while evidence regarding shivering and maternal satisfaction remains limited. This results in the current data not demonstrating a clear advantage of either vasopressor with respect to patient-reported outcomes.

Further research using standardised measures of maternal comfort and well-being is required to determine whether differences in agents pharmacologically translate into clinically meaningful benefits for patients. 

Neonatal outcomes

Neonatal well-being is a key consideration when selecting vasopressors for the prevention and treatment of spinal-induced hypotension during caesarean delivery. Historically, concerns regarding uteroplacental perfusion strongly influenced vasopressor choice, with ephedrine being favoured because pure α-adrenergic vasoconstrictors were thought to reduce uterine blood flow and compromise fetal oxygenation [5]. However, subsequent evidence demonstrated that phenylephrine was associated with more favourable fetal acid-base status than ephedrine, leading to its widespread adoption as the first-line vasopressor in obstetric anaesthesia [2,6]. As noradrenaline has emerged as a potential alternative, research has increasingly focused on whether its maternal haemodynamic advantages are achieved without compromising neonatal safety.

Umbilical Arterial pH and Fetal Acid-Base Status

Umbilical arterial pH is widely regarded as one of the most objective indicators of neonatal well-being following caesarean delivery. It reflects fetal acid-base status at birth and may provide a more sensitive assessment of fetal compromise than Apgar scores alone [6,19].

Ngan Kee et al. conducted one of the largest randomised controlled trials comparing phenylephrine and noradrenaline during caesarean delivery under spinal anaesthesia [19]. The authors demonstrated that noradrenaline was non-inferior to phenylephrine regarding umbilical arterial pH, with no clinically significant differences observed between groups. These findings were important because they suggested that the improved maternal haemodynamic profile associated with noradrenaline did not occur at the expense of fetal acid-base status.

Similar findings have been reported in several smaller randomised controlled trials. Vallejo et al. and Sharkey et al. reported comparable umbilical cord blood gas values between treatment groups. These studies support the view that noradrenaline can maintain fetal acid-base balance despite its vasoconstrictor effects [12,13].

More recently, Kang et al. performed a systematic review and meta-analysis specifically examining umbilical cord blood gas outcomes [17]. The authors found no significant differences in umbilical arterial pH, umbilical venous pH, base excess, or lactate concentrations between noradrenaline and phenylephrine. Similarly, Liu et al. concluded that neonatal acid-base outcomes were equivalent between the two vasopressors [15].

Despite these reassuring findings, several limitations should be acknowledged. Most included studies involved healthy women undergoing elective caesarean section and excluded pregnancies complicated by fetal growth restriction, placental insufficiency, severe pre-eclampsia, or fetal distress. As a result, current evidence primarily reflects low-risk obstetric populations. Furthermore, many studies were designed to detect maternal haemodynamic differences rather than neonatal outcomes, increasing the possibility of type II error. Therefore, these findings should be interpreted cautiously when considering higher-risk obstetric populations.

Umbilical Cord Blood Gas Parameters

In addition to umbilical arterial pH, several studies have examined other cord blood gas parameters, including base excess, bicarbonate concentration, carbon dioxide tension, and lactate levels. Together, these measures provide a broader assessment of fetal metabolic status and oxygenation at birth [17,19].

Randomised controlled trials have generally shown no significant differences in these parameters between phenylephrine and noradrenaline groups. Ngan Kee et al., Vallejo et al., and Sharkey et al. reported similar biochemical markers between groups [12,13,19], and these findings were supported by the meta-analysis conducted by Kang et al. [17].

Nevertheless, cord blood gas parameters represent short-term physiological outcomes measured immediately after birth. They provide limited insight into longer-term neonatal adaptation, neurological outcomes, or developmental consequences. Therefore, while current evidence is reassuring, it does not establish long-term safety.

Apgar Scores

Apgar scores remain one of the most commonly reported neonatal outcomes in studies evaluating obstetric vasopressors [15,17]. Although less sensitive than umbilical cord blood gas analysis, they provide a simple and widely accepted measure of neonatal adaptation immediately after birth.

Ngan Kee et al., Vallejo et al., and Sharkey et al. reported no significant differences in one-minute or five-minute Apgar scores between phenylephrine and noradrenaline groups [12,13,19]. These findings have also been supported by systematic reviews and meta-analyses [15,17].

However, Apgar scores have recognised limitations as measures of fetal well-being, as they are influenced by multiple factors unrelated to vasopressor choice, including gestational age, anaesthetic technique, maternal medications, and subjective clinician assessment. They may also fail to detect subtle physiological changes. Consequently, Apgar scores should be interpreted alongside more objective measures such as umbilical cord blood gas analysis.

Neonatal Morbidity and Clinical Outcomes

Although umbilical cord blood gas values and Apgar scores are frequently reported, clinically meaningful neonatal outcomes, such as neonatal intensive care unit admission, respiratory complications, need for resuscitation, and neonatal morbidity, are reported less consistently.

Available studies have not demonstrated clear differences in these outcomes between phenylephrine and noradrenaline. Ngan Kee et al. reported similar rates of neonatal intervention and adverse neonatal events between groups [19]. Similarly, pooled analyses conducted by Liu et al. and Ahmed et al. found no evidence that either vasopressor increased neonatal morbidity [15,16].

However, these findings should be interpreted with caution. Serious neonatal complications are relatively uncommon in elective caesarean sections, meaning most randomised trials are substantially underpowered to detect meaningful differences in rare adverse outcomes. Furthermore, neonatal morbidity is often reported as a secondary outcome and may not be systematically assessed. Therefore, the absence of observed differences does not necessarily establish definitive equivalence [15,16].

A further limitation of the literature is the near-complete absence of long-term neonatal follow-up. No major randomised trial has evaluated neurodevelopmental outcomes, infant growth, or long-term health following exposure to either vasopressor [15,17]. Consequently, current evidence only supports short-term neonatal safety.

Overall Comparison of Neonatal Outcomes

Overall, the neonatal literature suggests that noradrenaline and phenylephrine have broadly comparable short-term safety profiles during caesarean delivery under spinal anaesthesia. This conclusion is supported by findings across umbilical arterial pH, cord blood gas parameters, Apgar scores, and reported neonatal morbidity.

Nevertheless, important gaps remain. Most studies are derived from low-risk elective caesarean sections and were not designed to assess rare neonatal adverse events or long-term developmental outcomes. Therefore, while current evidence is reassuring, further research is needed before definitive conclusions can be drawn regarding neonatal superiority or long-term safety.

From a clinical perspective, the neonatal evidence supports equivalence rather than superiority. This is important because phenylephrine replaced ephedrine largely because of demonstrated neonatal acid-base advantages. In contrast, the potential benefits of noradrenaline appear to relate mainly to maternal haemodynamic outcomes, while neonatal outcomes do not currently show a clear advantage over phenylephrine.

Summary of Key Contemporary Evidence Comparing Phenylephrine and Noradrenaline

Table 1 presents a summary of the key contemporary evidence comparing phenylephrine and noradrenaline.

**Table 1:** Summary of Key Contemporary Evidence Comparing Phenylephrine and Noradrenaline RCT: randomised controlled trials; TSA: trial sequential analysis

Author & Year	Study Cohort & Design	Dosing Strategy	Primary Maternal Hemodynamic Findings	Primary Neonatal Findings
Lee et al. (2002) [5]	Quantitative systematic review; 7 RCTs (n=292 healthy, non-laboring patients)	Pooled analysis of varied ephedrine vs. phenylephrine protocols	Phenylephrine was as effective as ephedrine in maintaining maternal blood pressure	Challenged old assumptions; found phenylephrine was not associated with adverse neonatal outcomes
Ngan Kee et al. (2008) [6]	Randomized, double-blind RCT; n=125 healthy elective cases	Infusion combinations of phenylephrine and ephedrine at varying concentration ratios	Provided effective maternal blood pressure control during elective cases.	Phenylephrine-based regimens resulted in higher umbilical arterial pH and less fetal acidosis
Dyer et al. (2009) [9]	Randomized, double-blind RCT; n=43 healthy elective cases	Intermittent IV boluses comparing ephedrine, phenylephrine, and phenylephrine + oxytocin	Demonstrated that phenylephrine effectively restored blood pressure but caused reductions in maternal heart rate and cardiac output via reflex bradycardia	Favorable immediate outcomes, but raised initial concerns over macrovascular stability versus microvascular organ perfusion
Ngan Kee et al. (2015) [10]	n=104, healthy elective patients; double-blind RCT	Continuous infusion: noradrenaline 0.05 μg/kg/min vs. phenylephrine 0.1 μg/kg/min	Significantly fewer episodes of bradycardia; superior preservation of cardiac output in the noradrenaline arm.	No significant differences in umbilical blood gases or immediate fetal profiles
Ngan Kee (2017) [11]	Randomized, double-blind dose-response trial; n=180 healthy elective cases	Random-allocation graded IV bolus doses of noradrenaline vs. phenylephrine to determine potency	Calculated an approximate potency ratio of 13:1, proving that noradrenaline achieves target vasopressor thresholds at lower absolute doses	Primary focus was on microvascular/pharmacological potency metrics rather than tracking long-term clinical safety profiles
Vallejo et al. (2017) [12]	n =85, healthy elective patients; open-label RCT	Continuous infusion: noradrenaline 4 μg/ min vs. phenylephrine 100 μg /min	Equivalent efficacy in preventing hypotension; similar requirements for rescue vasopressor intervention	Comparable umbilical arterial pH and base-excess measurements
Sharkey et al. (2019) [13]	n =112, healthy elective patients; double-blind RCT	Intermittent boluses: noradrenaline 6 μg vs. phenylephrine 100 μg	Identical efficacy in treating breakthrough hypotension; lower incidence of transient maternal bradycardia	Equivalent 1-minute and 5-minute Apgar scores across both arms
Cho et al. (2020) [14]	n =56, healthy elective patients; double-blind RCT	Intermittent boluses: noradrenaline 5 μg vs. phenylephrine 100 μg	The noradrenaline group demonstrated higher heart rates and better-preserved non-invasive cardiac output indices	Broadly equivalent immediate neonatal metabolic profiles
Ngan Kee et al. (2020) [18]	Multi-center, parallel-group non-inferiority double-blind RCT; n=668 enrolled (664 completed), elective and non-elective cases.	Institutional standardized protocol dosing	Confirmed maternal hemodynamic baseline equivalence across a massive clinical sample size	Confirmed definitive non-inferiority of noradrenaline via objective umbilical arterial pH
Liu et al. (2022) [15]	Systematic review and meta-analysis with TSA; 13 RCTs (n=1152 prophylactic cases)	Pooled analysis of varied prophylactic noradrenaline vs. phenylephrine regimens	Confirmed a lower incidence of maternal bradycardia with noradrenaline but identified high clinical heterogeneity in baseline trial definitions	Confirmed equivalent fetal acid-base and safety profiles across all pooled trial datasets
Ahmed et al. (2024) [16]	Systematic review and meta-analysis; pooled clinical trials focusing on obstetric patients (including preeclamptic subsets)	Pooled analysis of noradrenaline vs. phenylephrine administration for post-spinal hypotension	Demonstrated equal efficacy in blood pressure maintenance; noted a potential reduction in reactive maternal hypertension with noradrenaline	Documented comparable short-term neonatal morbidity, stable umbilical safety, and safe Apgar thresholds between groups
Kang et al. (2024) [17]	Systematic review and meta-analysis; 17 RCTs (n=1528 obstetric patients)	Global pooled analysis comparing the clinical outcomes of noradrenaline versus phenylephrine	Supported the ongoing narrative of maternal hemodynamic equivalence for target macrovascular blood pressure control	Explicitly demonstrated zero statistical difference in umbilical arterial pH, venous pH, base excess, or lactate levels

Current guidelines and clinical practice

Clinical guidelines are important because they determine how evidence is translated into routine obstetric anaesthetic practice [2,20,21]. Although recent trials increasingly support noradrenaline as a safe and effective alternative to phenylephrine, most formal guidance continues to favour phenylephrine as the first-line vasopressor [10,12,13,15,17,19]. This section compares current guideline recommendations with the emerging evidence base and the challenges of incorporating emerging evidence into established clinical practice.

International Consensus Statement on Vasopressor Management

The most widely cited guidance on vasopressor use during caesarean section under spinal anaesthesia is the international consensus statement by Kinsella et al. [2]. This statement recommends maintaining maternal systolic arterial pressure at ≥ 90% of baseline and avoiding a fall below 80% of baseline. It also recommends the use of prophylactic vasopressor infusion, started immediately after intrathecal injection, rather than waiting for hypotension to occur. Phenylephrine is recommended as the preferred first-line vasopressor, largely because of its established efficacy in maintaining maternal blood pressure and its favourable fetal acid-base profile compared with ephedrine [2].

The evidence supporting this recommendation comes from earlier studies showing that ephedrine was associated with lower umbilical arterial pH and greater fetal metabolic effects than phenylephrine [5,7]. However, this guidance was published before several important noradrenaline studies and recent meta-analyses became available. Therefore, although it remains influential, it provides limited guidance regarding the role of noradrenaline [2].

Association of Anaesthetists Guidance

The Association of Anaesthetists published and disseminated the 2018 international consensus guidance, which has strongly influenced UK obstetric anaesthetic practice. Phenylephrine is presented as the standard first-line vasopressor, with ephedrine reserved for selected situations, particularly where maternal heart rate is low [2].

This approach is supported by phenylephrine's predictable α1-mediated vasoconstriction, effective blood pressure control, and favourable fetal acid-base outcomes compared with ephedrine [5,6]. However, phenylephrine may cause reflex bradycardia and reduced cardiac output in some patients [9,10]. Although these haemodynamic effects have not been consistently linked to adverse maternal or neonatal outcomes, they have contributed to growing interest in alternative vasopressors, particularly noradrenaline, which may better preserve maternal heart rate and cardiac output.

Southeast Asian Consensus Statement

A more recent Southeast Asian consensus statement also supports proactive management of maternal hypotension during caesarean section under spinal anaesthesia. Similar to the international consensus statement, it supports phenylephrine as the first-line vasopressor and emphasises preventing hypotension rather than simply treating it after it occurs [20].

This consensus is useful because it considers implementation issues such as drug availability, cost, prefilled syringes, and patient safety systems. However, it still largely supports phenylephrine-based practice rather than fully endorsing noradrenaline as first-line therapy. As a result, the statement highlights the continuing gap between emerging research and formal guideline adoption [20].

National Institute for Health and Care Excellence (NICE) Caesarean Birth Guidance

NICE guidance on caesarean birth focuses more broadly on safe caesarean delivery, anaesthetic choice, and maternal safety rather than providing detailed vasopressor protocols. NICE recommends regional anaesthesia for most caesarean births because it avoids many of the risks associated with general anaesthesia and allows the mother to remain awake during delivery [21]. Although NICE does not directly compare phenylephrine and noradrenaline, it supports the broader principle that hypotension associated with regional anaesthesia should be anticipated and treated promptly.

The limitation of NICE guidance for this review is that it is not primarily a vasopressor guideline. Therefore, this offers limited insight into the comparative roles of vasopressors but remains important for understanding the wider framework of contemporary obstetric anaesthetic practice.

Society of Obstetric Anesthesia and Perinatology

The Society for Obstetric Anaesthesia and Perinatology does not have a single universally cited vasopressor guideline equivalent to the Kinsella et al. [2] consensus statement. However, contemporary North American obstetric anaesthetic practice generally aligns with the same evidence base: phenylephrine remains widely used because of its strong evidence for blood pressure control and neonatal acid-base safety, while noradrenaline is increasingly discussed as an alternative because of its more favourable maternal haemodynamic profile.

This reflects evidence from Ngan Kee et al. [10], Vallejo et al. [12], Sharkey et al. [13], and Ngan Kee et al. [19], which suggests that noradrenaline provides similar blood pressure control and neonatal outcomes to phenylephrine while reducing bradycardia and better preserving cardiac output. Although noradrenaline is attracting increasing interest in clinical practice, phenylephrine continues to be regarded as the standard against which newer vasopressor strategies are compared.

Overall Comparison of Current Guidelines

Overall, current guidance remains more supportive of phenylephrine than noradrenaline. The international consensus statement and Southeast Asian consensus both recommend phenylephrine as the first-line vasopressor for spinal-induced hypotension during caesarean section [2,20]. This is mainly because phenylephrine has a longer history of use, a larger evidence base, predictable efficacy, and favourable neonatal acid-base outcomes compared with ephedrine.

However, newer evidence increasingly supports noradrenaline as a credible alternative. Randomised trials and meta-analyses show that noradrenaline provides comparable blood pressure control and neonatal outcomes while reducing bradycardia and better preserving maternal cardiac output [10,13,15,17,19]. Therefore, the apparent discrepancy between guidelines and emerging evidence does not reflect contradiction, but rather differences in the maturity of the underlying evidence base [2,15,17]. They prioritise established safety and implementation simplicity, whereas recent research focuses on physiological advantages.

The main limitation of current guidelines is that they have not fully incorporated the latest noradrenaline evidence. Conversely, the limitation of the noradrenaline evidence is that it remains less mature than the phenylephrine evidence and is mostly derived from healthy women undergoing elective caesarean delivery. Therefore, phenylephrine remains the guideline-supported first-line agent, while noradrenaline should currently be interpreted as a promising alternative rather than a definitive replacement.

Discussion

This review demonstrates that both phenylephrine and noradrenaline are effective vasopressors for the prevention and treatment of spinal-induced hypotension during caesarean section. Current evidence consistently shows that both agents provide reliable blood pressure control and are associated with comparable neonatal outcomes [15-17,19]. The principal difference between the two vasopressors relates to maternal haemodynamic effects [10,12,13,15], with noradrenaline demonstrating better preservation of heart rate and cardiac output and a lower incidence of bradycardia.

Despite these findings, phenylephrine remains the guideline-supported first-line vasopressor in contemporary obstetric anaesthetic practice. This is reflected in the International Consensus Statement on the Management of Hypotension with Vasopressors During Caesarean Section Under Spinal Anaesthesia, which recommends phenylephrine based on its extensive evidence base, established profile, and favourable neonatal outcome [2]. The continued preference for phenylephrine highlights an important part of evidence-based practice where changes in clinical guidelines depend not only on the benefits of the evidence but also on the consistency and generalisability.

A key finding of this review is the apparent discrepancy between emerging research and current guideline recommendations. Recent randomised controlled trials and meta-analyses suggest that noradrenaline provides similar blood pressure control and neonatal safety while offering improved haemodynamic stability [10,15,17]. However, these advantages are demonstrated primarily through surrogate physiological outcomes [10,12,13,15,16], including heart rate, cardiac output, and incidence of bradycardia. In contrast, clinically important outcomes, such as maternal morbidity, neonatal complications, intensive care admissions, and long-term neonatal well-being, have shown little evidence of meaningful differences between vasopressor strategies. Therefore, while noradrenaline may be physiologically advantageous, its clinical superiority remains uncertain [15-17].

Interpretation of available findings is limited as most studies have been conducted in healthy women undergoing elective caesarean delivery. High-risk groups, including women with severe pre-eclampsia, cardiovascular disease, obesity, or fetal compromise, remain underrepresented. Because clinical guidelines must be applicable across diverse patient populations, this lack of evidence may be insufficient to justify widespread changes in practice [2,20].

Future research

Future research should determine whether the haemodynamic advantages of noradrenaline translate into meaningful clinical benefits. Larger multicentre randomised controlled trials are needed to assess maternal morbidity, patient satisfaction, recovery outcomes, and high-risk obstetric populations, where preservation of cardiac output may be particularly relevant. Greater standardisation of dosing protocols and definitions of hypotension would also improve comparison between studies. Although short-term neonatal outcomes appear comparable between phenylephrine and noradrenaline, long-term neonatal follow-up remains limited.

## Conclusions

Overall, current evidence supports noradrenaline as a safe and effective alternative to phenylephrine, with broadly comparable efficacy rather than clear superiority. However, the evidence is not yet sufficient to justify a change in guideline recommendations. Phenylephrine should therefore remain the guideline-supported first-line vasopressor. For clinicians, noradrenaline may be considered when preservation of maternal heart rate and cardiac output is clinically important, particularly in selected patients where these haemodynamic effects may influence management.

## References

[1] Mercier FJ, Augè M, Hoffmann C, Fischer C, Le Gouez A (2013). Maternal hypotension during spinal anesthesia for caesarean delivery. Minerva Anestesiol.

[2] Kinsella SM, Carvalho B, Dyer RA (2018). International consensus statement on the management of hypotension with vasopressors during caesarean section under spinal anaesthesia. Anaesthesia.

[3] Klöhr S, Roth R, Hofmann T, Rossaint R, Heesen M (2010). Definitions of hypotension after spinal anaesthesia for caesarean section: literature search and application to parturients. Acta Anaesthesiol Scand.

[4] Butwick AJ, Columb MO, Carvalho B (2015). Preventing spinal hypotension during caesarean delivery: what is the latest?. Br J Anaesth.

[5] Lee A, Ngan Kee WD, Gin T (2002). A quantitative, systematic review of randomized controlled trials of ephedrine versus phenylephrine for the management of hypotension during spinal anesthesia for cesarean delivery. Anesth Analg.

[6] Ngan Kee WD, Lee A, Khaw KS, Ng FF, Karmakar MK, Gin T (2008). A randomized double-blinded comparison of phenylephrine and ephedrine infusion combinations to maintain blood pressure during spinal anesthesia for cesarean delivery: the effects on fetal acid-base status and hemodynamic control. Anesth Analg.

[7] Ngan Kee WD, Khaw KS, Lau TK, Ng FF, Chui K, Ng KL (2008). Randomised double-blinded comparison of phenylephrine vs ephedrine for maintaining blood pressure during spinal anaesthesia for non-elective Caesarean section*. Anaesthesia.

[8] Heesen M, Rijs K, Hilber N, Ngan Kee WD, Rossaint R, van der Marel C, Klimek M (2019). Ephedrine versus phenylephrine as a vasopressor for spinal anaesthesia-induced hypotension in parturients undergoing high-risk caesarean section: meta-analysis, meta-regression and trial sequential analysis. Int J Obstet Anesth.

[9] Dyer RA, Reed AR, van Dyk D (2009). Hemodynamic effects of ephedrine, phenylephrine, and the coadministration of phenylephrine with oxytocin during spinal anesthesia for elective cesarean delivery. Anesthesiology.

[10] Ngan Kee WD, Lee SW, Ng FF, Tan PE, Khaw KS (2015). Randomized double-blinded comparison of norepinephrine and phenylephrine for maintenance of blood pressure during spinal anesthesia for cesarean delivery. Anesthesiology.

[11] Ngan Kee WD (2017). A random-allocation graded dose-response study of norepinephrine and phenylephrine for treating hypotension during spinal anesthesia for cesarean delivery. Anesthesiology.

[12] Vallejo MC, Attaallah AF, Elzamzamy OM (2017). An open-label randomized controlled clinical trial for comparison of continuous phenylephrine versus norepinephrine infusion in prevention of spinal hypotension during cesarean delivery. Int J Obstet Anesth.

[13] Sharkey AM, Siddiqui N, Downey K, Ye XY, Guevara J, Carvalho JC (2019). Comparison of intermittent intravenous boluses of phenylephrine and norepinephrine to prevent and treat spinal-induced hypotension in cesarean deliveries: randomized controlled trial. Anesth Analg.

[14] Cho WJ, Cho SY, Lee AR (2020). Systemic hemodynamic effects of norepinephrine versus phenylephrine in intermittent bolus doses during spinal anesthesia for cesarean delivery. Anesth Pain Med (Seoul).

[15] Liu P, He H, Zhang SS, Liang Y, Gao ZJ, Yuan H, Dong BH (2022). Comparative efficacy and safety of prophylactic norepinephrine and phenylephrine in spinal anesthesia for cesarean section: a systematic review and meta-analysis with trial sequential analysis. Front Pharmacol.

[16] Ahmed S, Ahmad E, Fatima E (2024). Efficacy and safety of norepinephrine versus phenylephrine for post-spinal hypotension in preeclamptic patients: a systematic review and meta-analysis. Eur J Obstet Gynecol Reprod Biol.

[17] Kang H, Sung TY, Jee YS, Kwon W, Cho SA, Ahn S, Cho CK (2024). A comparison of norepinephrine versus phenylephrine to prevent hypotension after spinal anesthesia for cesarean section: systematic review and meta-analysis. J Pers Med.

[18] Tao W, Xie Y, Bao J, Ding W, Zhang Y, Hu X (2024). Comparison of the effects of norepinephrine and phenylephrine on shivering and hypothermia in patients undergoing caesarean section under spinal anaesthesia at a tertiary hospital in China: a randomised, double-blind, controlled trial protocol. BMJ Open.

[19] Ngan Kee WD, Lee SW, Ng FF, Lee A (2020). Norepinephrine or phenylephrine during spinal anaesthesia for caesarean delivery: a randomised double-blind pragmatic non-inferiority study of neonatal outcome. Br J Anaesth.

[20] Herbosa GA, Tho NN, Gapay AA, Lorsomradee S, Thang CQ (2022). Consensus on the Southeast Asian management of hypotension using vasopressors and adjunct modalities during cesarean section under spinal anesthesia. J Anesth Analg Crit Care.

[21] (2026). NICE guidelines: caesarean birth. https://www.nice.org.uk/guidance/ng192.

